# Comment on “Intrapartum Intrauterine Fetal Demise with Normal Umbilical Cord Blood Gas Values at Birth”

**DOI:** 10.1155/2015/191426

**Published:** 2015-04-09

**Authors:** Jeffrey J. Pomerance

**Affiliations:** Pediatrics, UCLA, 351 W. Northridge Avenue, Glendora, CA 91741-2036, USA

I read with interest “Intrapartum Intrauterine Fetal Demise with Normal Umbilical Cord Blood Gas Values at Birth,” by Michael D. Benson [1]. I am most pleased that Dr. Benson was willing to share the fetal monitor strips which show the following highlights:

- Initial baseline fetal heart rate of 140 beats per minute (bpm) with normal variability and accelerations.
- Intermittent variable and prolonged decelerations.
- Beginning three hours prior to delivery, increasing frequency and severity of variable decelerations associated with every instance of maternal pushing.
- Fifty minutes prior to delivery, a prolonged deceleration of eight minutes followed by a fetal heart rate of 160–190 bpm.
- Fetal heart rate tracing ends 27 minutes prior to delivery at 190 bpm.

Apgar scores were 0^1^/0^5^/0^10^/0^20^.

Cord gases were reported as (base excesses transposed in Table 1, correctly identified in text)

- vein: 7.27/48/<25/−10 (pH/PCO_2_/PO_2_/BE);
- artery: 7.13/64/<25/−5.

Although the title of this report calls the cord gas results “normal,” they are not. The arterial umbilical cord gas base excess of minus 10 is mildly abnormal [2, 3]. Further, the venoarterial pH difference of 0.14 is widened [4]. Terminal fetal bradycardia with widened pH differences is associated with both cord compression (common) [5, 6] and chronic fetal heart failure (rare) [7]. Typically, in chronic fetal heart failure, fetal heart rate variability is decreased throughout the fetal heart rate tracing and variable decelerations are not associated with the tracing. Therefore, this second option seems highly unlikely. The first option, cord compression with terminal fetal bradycardia, seems quite likely as there was evidence of cord compression (increasing frequency and severity of variable decelerations) and the presence of a widened pH difference in the cord gases. In cord occlusion, a widened pH difference is due to temporary restoration of umbilical cord arterial blood flow secondary to fetal hypertension. Although no evidence of cord compression was found at the time of cesarean delivery, occult cord compression is only identified if the cord happens to be directly below the uterine incision, as it is in only the minority of occurrences. The severe variable decelerations attest to the presence of impingement on the cord. A report [8] of cord blood gases associated with cord prolapse in a stillborn baby found values extremely similar (vein: 7.24/55/20/−5; artery: 7.10/71/8/−10) to those reported by Benson.

Cord occlusion with terminal fetal bradycardia and near-normal cord gases may occur in the absence of a widened venoarterial pH difference, although this finding is quite rare [9]. In order to establish cord compression with terminal fetal bradycardia as the likely etiology of neonatal depression in the absence of a widened venoarterial pH difference, one must demonstrate preceding evidence of a vulnerable cord, the presence of normal or near-normal venous and arterial cord gases, and a postresuscitation blood gas that has a much worse base deficit.

Cord gases only reflect uteroplacental status (vein) and uteroplacental-fetal status (arteries) while blood flow continues.
